# 3-{(*E*)-[1-(2-Hy­droxy­phen­yl)ethyl­idene]amino}-1-(2-methyl­phen­yl)thio­urea

**DOI:** 10.1107/S1600536811013729

**Published:** 2011-04-16

**Authors:** Md. Abdus Salam, Md. Abu Affan, Mohd. Razip Asaruddin, Seik Weng Ng, Edward R. T. Tiekink

**Affiliations:** a Faculty of Resource Science and Technology, Universiti Malaysia Sarawak, 94300 Kota Samarahan, Sarawak, Malaysia; b Department of Chemistry, University of Malaya, 50603 Kuala Lumpur, Malaysia

## Abstract

In the title thio­urea derivative, C_16_H_17_N_3_OS, the hy­droxy- and methyl-substituted benzene rings form dihedral angles of 9.62 (12) and 55.69 (6)°, respectively, with the central CN_3_S chromophore (r.m.s. deviation = 0.0117 Å). An intra­molecular O—H⋯N hydrogen bond ensures the coplanarity of the central atoms. The H atoms of the NH groups are *syn* and the conformation about the N=C double bond [1.295 (4) Å] is *E*. In the crystal, helical supra­molecular chains sustained primarily by N—H⋯S hydrogen bonds are found. Additional stabilization is provided by C—H⋯π and π–π [ring centroid(hy­droxy­benzene)⋯ring centroid(methyl­benzene) = 3.8524 (18) Å] inter­actions.

## Related literature

For pharmaceutical applications of thio­ruea derivatives, see: Venkatachalam *et al.* (2004 ▶); Bruce *et al.* (2007 ▶). For related thio­urea structures, see: Normaya *et al.* (2011 ▶); Salam *et al.* (2011 ▶); Dzulkifli *et al.* (2011 ▶).
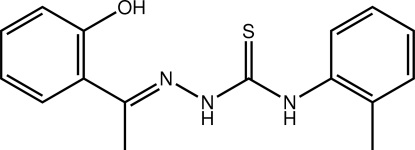


## Experimental

### 

#### Crystal data

C_16_H_17_N_3_OS
*M* *_r_* = 299.39
Monoclinic, 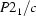

*a* = 14.6966 (8) Å
*b* = 7.3586 (4) Å
*c* = 14.0926 (8) Å
β = 94.358 (5)°
*V* = 1519.66 (15) Å^3^
*Z* = 4
Mo *K*α radiation
μ = 0.22 mm^−1^
*T* = 100 K
0.30 × 0.10 × 0.05 mm

#### Data collection

Agilent Supernova Dual diffractometer with an Atlas detector
Absorption correction: multi-scan (*CrysAlis PRO*; Agilent, 2010 ▶) *T*_min_ = 0.419, *T*_max_ = 1.000
7614 measured reflections
3375 independent reflections
2094 reflections with *I* > 2σ(*I*)
*R*_int_ = 0.066

#### Refinement

*R*[*F* ^2^ > 2σ(*F* ^2^)] = 0.060
*wR*(*F* ^2^) = 0.174
*S* = 1.00
3375 reflections
201 parameters
3 restraints
H atoms treated by a mixture of independent and constrained refinement
Δρ_max_ = 0.34 e Å^−3^
Δρ_min_ = −0.34 e Å^−3^

### 

Data collection: *CrysAlis PRO* (Agilent, 2010 ▶); cell refinement: *CrysAlis PRO*; data reduction: *CrysAlis PRO*; program(s) used to solve structure: *SHELXS97* (Sheldrick, 2008 ▶); program(s) used to refine structure: *SHELXL97* (Sheldrick, 2008 ▶); molecular graphics: *ORTEP-3* (Farrugia, 1997 ▶) and *DIAMOND* (Brandenburg, 2006 ▶); software used to prepare material for publication: *publCIF* (Westrip, 2010 ▶).

## Supplementary Material

Crystal structure: contains datablocks global, I. DOI: 10.1107/S1600536811013729/hg5025sup1.cif

Structure factors: contains datablocks I. DOI: 10.1107/S1600536811013729/hg5025Isup2.hkl

Additional supplementary materials:  crystallographic information; 3D view; checkCIF report

## Figures and Tables

**Table 1:** Hydrogen-bond geometry (Å, °) *Cg*1 is the centroid of the C10–C15 ring.

*D*—H⋯*A*	*D*—H	H⋯*A*	*D*⋯*A*	*D*—H⋯*A*
O1—H1o⋯N1	0.84 (1)	1.81 (2)	2.551 (3)	145 (3)
N2—H2n⋯S1^i^	0.88 (1)	2.51 (2)	3.323 (2)	154 (3)
N3—H3n⋯S1^i^	0.88 (1)	2.49 (2)	3.286 (3)	151 (2)
C8—H8b⋯*Cg*1^i^	0.98	2.59	3.501 (3)	155

Symmetry code: (i) 
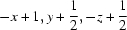
.

**Fig. 1.**
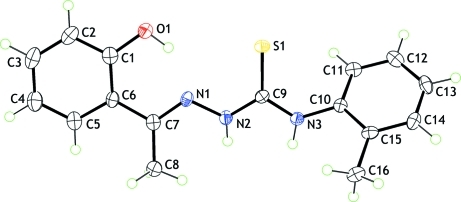
The molecular structure of (I) showing the atom-labelling scheme and displacement ellipsoids at the 50% probability level.

**Fig. 2.**
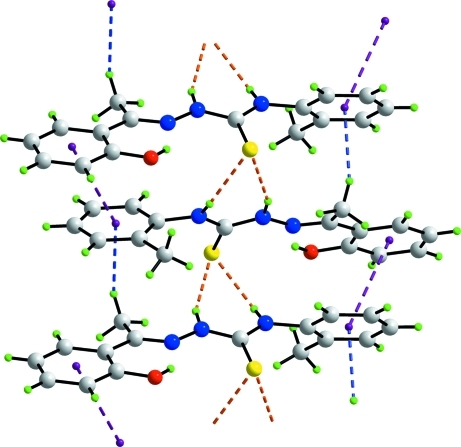
A view of the helical supramolecular chain aligned along the b axis in (I). The N—H···S hydrogen bonds are shown as orange dashed lines. Further stabilization to the chain is provided by C—H···π and π–π interactions, shown as blue and purple dashed lines, respectively.

**Fig. 3.**
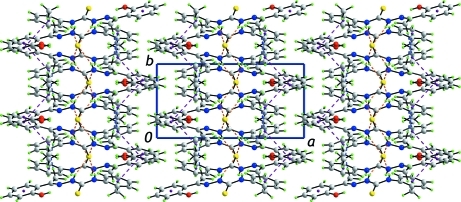
A view in projection down the c axis of the crystal packing in (I) showing the staking of layers comprising the helical supramolecular chains shown in Fig. 2. The O—H···O and N—H···S hydrogen bonds (orange), and C—H···π (blue) and π–π (purple) interactions are shown as dashed lines.

## supplementary crystallographic information

### Comment

In continuation of structural investigations into the conformation and hydrogen bonding patterns in thiourea derivatives (Normaya *et al.* 2011; Salam *et al.*, 2011; Dzulkifli *et al.*, 2011), and also motivated by their pharmacological potential (Venkatachalam *et al.* 2004; Bruce *et al.*, 2007), the title compound, (I), was investigated.

With respect to the planar (r.m.s. = 0.0117 Å) central CN_3_S chromophore in (I), Fig. 1, the OH– and Me-benzene rings are twisted as seen in the respective dihedral angles of 9.62 (12) and 55.69 (6) °. The almost co-planarity of the central atoms is ascribed to the formation of an intramolecular hydroxyl-*O*—*H*···*N*-imine hydrogen bond, Table 1. The H atoms of the NH groups are *syn*, and the conformation about the N1═C7 double bond [1.295 (4) Å] is *E*. The *syn* arrangement in (I) contrast the *anti* arrangement often seen in such derivatives but is readily explained in terms of the intramolecular *O*—*H*···*N*-imine hydrogen bond in (I) by contrast to the normally observed intramolecular *N*—*H*···*N*-imine hydrogen bond (Normaya *et al.* 2011; Salam *et al.*, 2011; Dzulkifli *et al.*, 2011).

Helical supramolecular chains along the *b* axis dominate the crystal packing, Fig. 2 and Table 1. These arise as a result of the thione-S interacting with both N—H atoms of a neighbouring molecule thereby forming six-membered hydrogen bond mediated rings. Chains are stabilized by C—H..π, Table 1, and π–π [ring centroid(C1···C6)···ring centroid(C10···C15)^i^ = 3.8524 (18) Å, dihedral angle = 2.37 (15) ° for *i*: 1 - *x*, -1/2 + *y*, 1/2 - *z*] interactions, Fig. 3.

### Experimental

2-Methylphenylisothiocyanate (0.746 g, 5 mmol) and hydrazine hydrate (0.253 g, 5 mmol), each dissolved in 10 ml e thanol, were mixed with constant stirring. The stirring was continued for 30 min and the white product formed was washed with ethanol and dried *in vacuo*. A solution of the isolated 2-methylphenylthiosemicarbazide (0.540 g, 3 mmol) in 10 ml me thanol was then refluxed with a methanolic solution of 2-hydroxyacetophenone (0.408 g, 3 mmol) for 5 h after adding 1–2 drops of glacial acetic acid. On cooling, the solution to room temperature, a light-yellow powder separated and washed with methanol. The powder was recrystallized from methanol and dried *in vacuo* over silica gel. (*M*.pt. 451–453 K. Yield 0.740 g (78%). Elemental analysis: Calc. for C_16_H_17_N_3_OS: C, 64.21; H, 5.73; N, 14.04%. Found: C, 64.17; H, 5.67; N, 14.01%. FT—IR (KBr, cm^-1^) ν_max_: 3175 (m, OH), 3000 (s, NH), 1583 (w, C═N), 943 (m, N—N), 1371, 861 (w, C═S).

### Refinement

Carbon-bound H-atoms were placed in calculated positions (C–H = 0.98 to 1.00 Å) and were included in the refinement in the riding model approximation, with *U*_iso_(H) set to 1.2–1.5*U*_eq_(C). The O– and N-bound H-atoms were located in a difference Fourier map and were refined with distance restraints of O—H = 0.84±0.01 Å and N—H 0.88±0.01 Å, and with *U*_iso_(H) = *yU*_eq_(parent atom) for *y* = 1.5 (O) and 1.2 (N).

### Crystal data

**Table:**

C_16_H_17_N_3_OS	*F*(000) = 632
*M**_r_* = 299.39	*D*_x_ = 1.309 Mg m^−^^3^
Monoclinic, *P*2_1_/*c*	Mo *K*α radiation, λ = 0.71073 Å
Hall symbol: -P 2ybc	Cell parameters from 1905 reflections
*a* = 14.6966 (8) Å	θ = 2.8–29.3°
*b* = 7.3586 (4) Å	µ = 0.22 mm^−^^1^
*c* = 14.0926 (8) Å	*T* = 100 K
β = 94.358 (5)°	Prism, light-yellow
*V* = 1519.66 (15) Å^3^	0.30 × 0.10 × 0.05 mm
*Z* = 4	

### Data collection

**Table:**

Agilent Supernova Dual diffractometer with an Atlas detector	3375 independent reflections
Radiation source: SuperNova (Mo) X-ray Source	2094 reflections with *I* > 2σ(*I*)
Mirror	*R*_int_ = 0.066
Detector resolution: 10.4041 pixels mm^-1^	θ_max_ = 27.5°, θ_min_ = 2.8°
ω scans	*h* = −14→19
Absorption correction: multi-scan (*CrysAlis PRO*; Agilent, 2010)	*k* = −9→7
*T*_min_ = 0.419, *T*_max_ = 1.000	*l* = −18→15
7614 measured reflections	

### Refinement

**Table:**

Refinement on *F*^2^	Primary atom site location: structure-invariant direct methods
Least-squares matrix: full	Secondary atom site location: difference Fourier map
*R*[*F*^2^ > 2σ(*F*^2^)] = 0.060	Hydrogen site location: inferred from neighbouring sites
*wR*(*F*^2^) = 0.174	H atoms treated by a mixture of independent and constrained refinement
*S* = 1.00	*w* = 1/[σ^2^(*F*_o_^2^) + (0.0769*P*)^2^] where *P* = (*F*_o_^2^ + 2*F*_c_^2^)/3
3375 reflections	(Δ/σ)_max_ < 0.001
201 parameters	Δρ_max_ = 0.34 e Å^−^^3^
3 restraints	Δρ_min_ = −0.34 e Å^−^^3^

### Special details

**Table:**

Geometry. All s.u.'s (except the s.u. in the dihedral angle between two l.s. planes) are estimated using the full covariance matrix. The cell s.u.'s are taken into account individually in the estimation of s.u.'s in distances, angles and torsion angles; correlations between s.u.'s in cell parameters are only used when they are defined by crystal symmetry. An approximate (isotropic) treatment of cell s.u.'s is used for estimating s.u.'s involving l.s. planes.
Refinement. Refinement of *F*^2^ against ALL reflections. The weighted *R*-factor *wR* and goodness of fit *S* are based on *F*^2^, conventional *R*-factors *R* are based on *F*, with *F* set to zero for negative *F*^2^. The threshold expression of *F*^2^ > 2σ(*F*^2^) is used only for calculating *R*-factors(gt) *etc*. and is not relevant to the choice of reflections for refinement. *R*-factors based on *F*^2^ are statistically about twice as large as those based on *F*, and *R*- factors based on ALL data will be even larger.

### Fractional atomic coordinates and isotropic or equivalent isotropic displacement parameters (Å^2^)

**Table:**

	*x*	*y*	*z*	*U*_iso_*/*U*_eq_	
S1	0.46230 (5)	0.24817 (10)	0.38597 (5)	0.0188 (2)	
O1	0.21619 (14)	0.2307 (3)	0.31997 (15)	0.0250 (5)	
H1O	0.2693 (11)	0.261 (4)	0.308 (2)	0.038*	
N1	0.33770 (15)	0.3710 (3)	0.22261 (17)	0.0182 (6)	
N2	0.42781 (16)	0.4196 (3)	0.21980 (18)	0.0190 (6)	
H2N	0.4433 (19)	0.497 (3)	0.1764 (16)	0.023*	
N3	0.57409 (16)	0.4362 (3)	0.27942 (17)	0.0197 (6)	
H3N	0.5801 (19)	0.496 (4)	0.2259 (13)	0.024*	
C1	0.1592 (2)	0.2588 (4)	0.2410 (2)	0.0229 (7)	
C2	0.0685 (2)	0.2066 (5)	0.2458 (3)	0.0309 (8)	
H2	0.0496	0.1544	0.3027	0.037*	
C3	0.0062 (2)	0.2298 (5)	0.1692 (3)	0.0331 (9)	
H3A	−0.0553	0.1927	0.1732	0.040*	
C4	0.0327 (2)	0.3073 (5)	0.0857 (3)	0.0320 (8)	
H4	−0.0104	0.3236	0.0327	0.038*	
C5	0.1220 (2)	0.3603 (4)	0.0806 (2)	0.0260 (8)	
H5A	0.1396	0.4142	0.0236	0.031*	
C6	0.18777 (19)	0.3372 (4)	0.1568 (2)	0.0200 (7)	
C7	0.28345 (19)	0.3879 (4)	0.1464 (2)	0.0189 (7)	
C8	0.3131 (2)	0.4548 (4)	0.0530 (2)	0.0229 (7)	
H8A	0.3795	0.4432	0.0525	0.034*	
H8B	0.2957	0.5827	0.0445	0.034*	
H8C	0.2834	0.3824	0.0012	0.034*	
C9	0.48965 (19)	0.3751 (4)	0.2920 (2)	0.0174 (7)	
C10	0.65424 (19)	0.4166 (4)	0.3422 (2)	0.0172 (7)	
C11	0.6563 (2)	0.4776 (4)	0.4351 (2)	0.0201 (7)	
H11	0.6024	0.5242	0.4592	0.024*	
C12	0.7355 (2)	0.4712 (4)	0.4928 (2)	0.0252 (7)	
H12A	0.7364	0.5112	0.5570	0.030*	
C13	0.8147 (2)	0.4057 (4)	0.4568 (2)	0.0253 (7)	
H13	0.8703	0.4035	0.4960	0.030*	
C14	0.8128 (2)	0.3441 (4)	0.3647 (2)	0.0231 (7)	
H14	0.8671	0.2974	0.3414	0.028*	
C15	0.73287 (19)	0.3485 (4)	0.3047 (2)	0.0195 (7)	
C16	0.7318 (2)	0.2841 (4)	0.2034 (2)	0.0260 (8)	
H16A	0.7815	0.1969	0.1974	0.039*	
H16B	0.7401	0.3882	0.1615	0.039*	
H16C	0.6733	0.2253	0.1851	0.039*	

### Atomic displacement parameters (Å^2^)

**Table:**

	*U*^11^	*U*^22^	*U*^33^	*U*^12^	*U*^13^	*U*^23^
S1	0.0225 (4)	0.0163 (4)	0.0179 (4)	−0.0009 (3)	0.0039 (3)	−0.0001 (3)
O1	0.0214 (11)	0.0291 (13)	0.0247 (13)	−0.0029 (10)	0.0027 (10)	−0.0005 (10)
N1	0.0150 (12)	0.0148 (13)	0.0247 (15)	−0.0010 (10)	0.0019 (10)	−0.0010 (11)
N2	0.0179 (13)	0.0166 (14)	0.0222 (15)	−0.0023 (10)	0.0002 (11)	0.0044 (11)
N3	0.0187 (13)	0.0205 (14)	0.0199 (15)	−0.0006 (11)	0.0016 (11)	0.0052 (11)
C1	0.0245 (16)	0.0172 (16)	0.0269 (18)	0.0046 (13)	0.0018 (13)	−0.0085 (14)
C2	0.0234 (17)	0.030 (2)	0.040 (2)	−0.0028 (14)	0.0098 (15)	−0.0020 (16)
C3	0.0198 (17)	0.031 (2)	0.049 (2)	−0.0018 (14)	0.0039 (16)	−0.0077 (17)
C4	0.0248 (18)	0.0298 (19)	0.040 (2)	0.0018 (15)	−0.0039 (15)	0.0006 (17)
C5	0.0242 (17)	0.0207 (17)	0.032 (2)	0.0010 (14)	−0.0026 (14)	−0.0014 (15)
C6	0.0174 (15)	0.0158 (16)	0.0266 (18)	−0.0010 (12)	0.0003 (13)	−0.0037 (13)
C7	0.0209 (15)	0.0104 (15)	0.0250 (18)	0.0038 (12)	0.0004 (13)	−0.0030 (13)
C8	0.0248 (16)	0.0192 (17)	0.0247 (18)	−0.0002 (13)	0.0021 (13)	−0.0036 (14)
C9	0.0180 (15)	0.0151 (15)	0.0197 (16)	0.0034 (12)	0.0045 (12)	−0.0028 (13)
C10	0.0185 (15)	0.0126 (15)	0.0205 (17)	−0.0006 (12)	0.0014 (12)	0.0014 (12)
C11	0.0272 (16)	0.0095 (14)	0.0239 (18)	0.0015 (13)	0.0045 (13)	−0.0009 (13)
C12	0.0354 (19)	0.0158 (16)	0.0235 (18)	−0.0033 (14)	−0.0037 (14)	0.0013 (14)
C13	0.0249 (17)	0.0192 (17)	0.030 (2)	−0.0021 (13)	−0.0091 (14)	0.0055 (14)
C14	0.0171 (15)	0.0183 (16)	0.034 (2)	0.0001 (13)	0.0034 (13)	0.0026 (15)
C15	0.0262 (16)	0.0132 (15)	0.0200 (17)	−0.0058 (13)	0.0063 (13)	−0.0003 (13)
C16	0.0275 (17)	0.0240 (18)	0.0276 (19)	−0.0018 (14)	0.0094 (14)	−0.0020 (14)

### Geometric parameters (Å, °)

**Table:**

S1—C9	1.694 (3)	C6—C7	1.473 (4)
O1—C1	1.357 (4)	C7—C8	1.500 (4)
O1—H1O	0.842 (10)	C8—H8A	0.9800
N1—C7	1.295 (4)	C8—H8B	0.9800
N1—N2	1.375 (3)	C8—H8C	0.9800
N2—C9	1.352 (4)	C10—C11	1.383 (4)
N2—H2N	0.880 (10)	C10—C15	1.400 (4)
N3—C9	1.344 (3)	C11—C12	1.369 (4)
N3—C10	1.426 (4)	C11—H11	0.9500
N3—H3N	0.882 (10)	C12—C13	1.391 (4)
C1—C2	1.394 (4)	C12—H12A	0.9500
C1—C6	1.411 (4)	C13—C14	1.373 (4)
C2—C3	1.372 (5)	C13—H13	0.9500
C2—H2	0.9500	C14—C15	1.395 (4)
C3—C4	1.390 (5)	C14—H14	0.9500
C3—H3A	0.9500	C15—C16	1.503 (4)
C4—C5	1.376 (4)	C16—H16A	0.9800
C4—H4	0.9500	C16—H16B	0.9800
C5—C6	1.400 (4)	C16—H16C	0.9800
C5—H5A	0.9500		
			
C1—O1—H1O	108 (2)	H8A—C8—H8B	109.5
C7—N1—N2	119.0 (2)	C7—C8—H8C	109.5
C9—N2—N1	120.6 (2)	H8A—C8—H8C	109.5
C9—N2—H2N	119 (2)	H8B—C8—H8C	109.5
N1—N2—H2N	119.3 (19)	N3—C9—N2	113.2 (2)
C9—N3—C10	127.7 (2)	N3—C9—S1	124.3 (2)
C9—N3—H3N	115.6 (19)	N2—C9—S1	122.4 (2)
C10—N3—H3N	116.7 (19)	C11—C10—C15	121.0 (3)
O1—C1—C2	116.8 (3)	C11—C10—N3	120.9 (3)
O1—C1—C6	123.2 (3)	C15—C10—N3	117.9 (3)
C2—C1—C6	120.0 (3)	C12—C11—C10	120.5 (3)
C3—C2—C1	120.8 (3)	C12—C11—H11	119.7
C3—C2—H2	119.6	C10—C11—H11	119.7
C1—C2—H2	119.6	C11—C12—C13	119.5 (3)
C2—C3—C4	120.2 (3)	C11—C12—H12A	120.2
C2—C3—H3A	119.9	C13—C12—H12A	120.2
C4—C3—H3A	119.9	C14—C13—C12	120.1 (3)
C5—C4—C3	119.4 (3)	C14—C13—H13	120.0
C5—C4—H4	120.3	C12—C13—H13	120.0
C3—C4—H4	120.3	C13—C14—C15	121.5 (3)
C4—C5—C6	122.1 (3)	C13—C14—H14	119.3
C4—C5—H5A	119.0	C15—C14—H14	119.3
C6—C5—H5A	119.0	C14—C15—C10	117.4 (3)
C5—C6—C1	117.6 (3)	C14—C15—C16	121.1 (3)
C5—C6—C7	120.1 (3)	C10—C15—C16	121.5 (3)
C1—C6—C7	122.3 (3)	C15—C16—H16A	109.5
N1—C7—C6	115.1 (3)	C15—C16—H16B	109.5
N1—C7—C8	123.9 (3)	H16A—C16—H16B	109.5
C6—C7—C8	120.9 (3)	C15—C16—H16C	109.5
C7—C8—H8A	109.5	H16A—C16—H16C	109.5
C7—C8—H8B	109.5	H16B—C16—H16C	109.5
			
C7—N1—N2—C9	−168.5 (3)	C10—N3—C9—N2	178.6 (3)
O1—C1—C2—C3	−179.9 (3)	C10—N3—C9—S1	−3.6 (4)
C6—C1—C2—C3	0.3 (5)	N1—N2—C9—N3	−178.5 (2)
C1—C2—C3—C4	−0.5 (5)	N1—N2—C9—S1	3.7 (4)
C2—C3—C4—C5	0.1 (5)	C9—N3—C10—C11	−56.4 (4)
C3—C4—C5—C6	0.6 (5)	C9—N3—C10—C15	129.0 (3)
C4—C5—C6—C1	−0.8 (5)	C15—C10—C11—C12	−0.5 (4)
C4—C5—C6—C7	177.0 (3)	N3—C10—C11—C12	−174.9 (3)
O1—C1—C6—C5	−179.4 (3)	C10—C11—C12—C13	1.1 (4)
C2—C1—C6—C5	0.3 (4)	C11—C12—C13—C14	−1.5 (5)
O1—C1—C6—C7	2.8 (5)	C12—C13—C14—C15	1.2 (5)
C2—C1—C6—C7	−177.4 (3)	C13—C14—C15—C10	−0.6 (4)
N2—N1—C7—C6	−178.4 (2)	C13—C14—C15—C16	178.8 (3)
N2—N1—C7—C8	1.2 (4)	C11—C10—C15—C14	0.2 (4)
C5—C6—C7—N1	176.2 (3)	N3—C10—C15—C14	174.8 (3)
C1—C6—C7—N1	−6.1 (4)	C11—C10—C15—C16	−179.2 (3)
C5—C6—C7—C8	−3.4 (4)	N3—C10—C15—C16	−4.6 (4)
C1—C6—C7—C8	174.3 (3)		

### Hydrogen-bond geometry (Å, °)

**Table:**

*D*—H···*A*	*D*—H	H···*A*	*D*···*A*	*D*—H···*A*
O1—H1o···N1	0.842 (10)	1.81 (2)	2.551 (3)	145 (3)
N2—H2n···S1^i^	0.880 (10)	2.508 (16)	3.323 (2)	154 (3)
N3—H3n···S1^i^	0.882 (10)	2.485 (17)	3.286 (3)	151 (2)
C8—H8b···Cg1^i^	0.98	2.59	3.501 (3)	155

Symmetry codes: (i) −*x*+1, *y*+1/2, −*z*+1/2.
